# The m^6^A Reader YTHDF1 Accelerates the Osteogenesis of Bone Marrow Mesenchymal Stem Cells Partly via Activation of the Autophagy Signaling Pathway

**DOI:** 10.1155/2023/5563568

**Published:** 2023-07-25

**Authors:** Xiang Gao, Jian Wang, Yibo Wang, Weixu Li, Zhijun Pan

**Affiliations:** ^1^Department of Orthopedic Surgery, The Second Affiliated Hospital Zhejiang University School of Medicine, Hangzhou, Zhejiang, China; ^2^Orthopedics Research Institute of Zhejiang University, Hangzhou, Zhejiang, China; ^3^Key Laboratory of Motor System Disease Research and Precision Therapy of Zhejiang Province, Hangzhou, Zhejiang, China

## Abstract

N6-methyladenosine (m^6^A) mRNA methylation has emerged as an important player in many biological processes by regulating gene expression. As a crucial reader, YTHDF1 usually improves the translation efficiency of its target mRNAs. However, its roles in bone marrow mesenchymal stem cells (BMSCs) osteogenesis remain largely unknown. Here, we reported that YTHDF1, an m^6^A reader, is highly expressed during osteogenic differentiation of BMSCs. Upregulation of YTHDF1 increased osteogenic differentiation and proliferation capacity of BMSCs. Accordingly, downregulation of YTHDF1 inhibited osteogenic differentiation and proliferation capacity. Possible underlying mechanisms were explored, and analysis revealed that YTHDF1 could affect autophagy levels, thus regulating osteogenesis of BMSCs. In an *in vivo* study, we found that upregulation of YTHDF1 accelerates fracture healing with elevated bone volume fraction and trabecular thickness. Taken together, our study revealed that m^6^A reader YTHDF1 accelerates osteogenic differentiation of BMSCs partly via the autophagy signaling pathway. These findings reveal a previously unrecognized mechanism involved in the regulation of BMSCs osteogenesis, providing new ideas and target sites for the treatment of fracture.

## 1. Introduction

Bone marrow-derived mesenchymal stem cells (BMSCs) are multipotent stromal cells with the ability to differentiate into osteoblast, chondrocyte, and adipocytes both *in vitro* and *in vivo* [1, 2]. Osteogenesis of BMSCs is an important development event that results in bone formation [3]. Although osteogenic differentiation of BMSCs is well recognized to play a crucial role in maintaining normal bone repair and regeneration, the regulatory mechanism remains less defined.

*N* ^6^-methyladenosine (m^6^A) is one of the most prominent posttranscriptional modifications in eukaryotic mRNA. m^6^A mediates almost every aspect of mRNA metabolism, including splicing, export, turnover, translation, and regulates mRNA stability [4–6]. Recently, studies have shown that m^6^A modification plays a crucial role in bone metabolism [7]. For example, conditional knockout m^6^A methyltransferase *Mettl3* in mice induces the pathological characteristics of osteoporosis and resulted in impaired bone formation, incompetent osteogenic differentiation potential, and increased morrow adiposity. In addition, *Mettl3* overexpression protects mice from estrogen deficiency-induced osteoporosis [8]. FTO (fat-mass and obesity-associated protein) as a m^6^A demethylase is downregulated in patients with osteoporosis and promotes the osteogenesis of human MSCs [9]. ALKBH5 (alkB homolog 5), another m^6^A demethylase, negatively regulates osteogenic differentiation of MSCs by activating the PI3K/AKT pathway [10].

The fates of m^6^A modified mRNAs are dependent on m^6^A selective binding proteins (m^6^A reader proteins) [4]. YTHDF1 is the most versatile and powerful reader protein, and can enhance the translation efficiency of m^6^A modified mRNAs. As a crucial reader, YTHDF1 plays a central role in cellular functions by mediating protein translation of important genes or by affecting the expression of key factors involved in many important cell signaling pathways [11–15]. Autophagy is a well conserved lysosomal degradation pathway, which is known to be highly active during differentiation and development [16–18]. Previous study indicated that YTHDF1 is involved in biogenesis of autophagy [19]. Although a recent study revealed that YTHDF1 facilitated osteogenic differentiation *in vitro* and *in vivo* [20], little is known about the interaction between YTHDF1 and autophagy during osteogenesis of BMSCs. Here, we show that YTHDF1 promotes osteogenic differentiation of BMSCs, in part by the autophagy pathway, and accelerates the fracture healing process *in vivo*.

## 2. Materials and Methods

### 2.1. Cell Culture and Reagents

hBMSCs were purchased from Cyagen Biosciences Inc. (HUXMA-01001, Guangzhou, China). Cells were incubated in hBMSC growth medium (Cyagen Biosciences, Guangzhou, China) at 37°C in a cell incubator containing 5% CO_2_ with the medium being replaced every 3 days. Cells from passages three to seven were used in subsequent experiments.

Antibodies used for Western blotting including RUNX2 (ab236639, 1 : 1,000, Abcam), COL1A1 (AF1840, 1 : 1,000, Beyotime), SP7 (ab209484, 1 : 1,000, Abcam), YTHDF1 (ab252346, 1 : 1,000, Abcam), p62/SQSTM1 (AF5312, 1 : 1,000, Beyotime), LC3B (ab192890, 1 : 1,000, Abcam), active-*β*-Catenin (ab246504, 1 : 1,000, Abcam), and GAPDH (AF1186, 1 : 1,000, Beyotime). The small-interfering RNA- (siRNA-) mate transfection reagents were purchased from GenePharma (Shanghai, China).

### 2.2. Osteogenic Differentiation

For osteogenic induction, the hMSCs were seeded in 6-well plates and cultured in osteogenic induction medium (OIM) when the cells reached 70% confluence. OIM consisted of DMEM (1,000 mg/L glucose) with 10% FBS, 100 IU/mL penicillin, 100 IU/mL streptomycin, 100 nM dexamethasone, 50 *μ*M ascorbic acid, and 5 mM *β*-glycerophosphate (Sigma–Aldrich). The medium was changed every 3 days and continued to be induced to the desired time point.

### 2.3. Cell Viability Assay

For Cell Counting Kit-8 (CCK-8) assays, BMSCs were seeded in six pairs of duplicate wells from a 96-well plate at a density of 5 × 10^3^ cells per well. The absorbance was measured at 450 nm after 0, 1, 2, 3, and 4 days using a CCK-8 KIT (C0038, Beyotime, China).

The EdU assay was carried out using an EdU DNA Cell Proliferation Kit (C0075, Beyotime). In brief, after incubation with EdU for 2 hr, cells were fixed with 4% paraformaldehyde followed by staining with Click Dye Solution and Hoechst 33342. The samples were then observed under a fluorescence microscope (Leica, Wetzlar, Germany).

### 2.4. ALP Staining

ALP staining was performed with a BCIP/NBT Alkaline Phosphatase Color Development Kit (C3206, Beyotime). In summary, MSCs were inoculated in a 24-well plate and cultured in OIM when the cells reached 70% confluence. OIM was cultured for 5 days, cells were washed with PBS three times, and fixed in 4% paraformaldehyde for 20 min at RT. Washed with PBS three times, BCIP/NBT working solution (500 *µ*L) was placed in each well and incubated in the dark for ALP staining for 20 min. To quantify the ALP, the ALP activity was determined by the ALP Activity Assay Kit (P0321S, Beyotime). The stain was lysed with lysis buffer consisting of 20 mM tris-HCl (pH 7.5), 1% triton X-100, and 150 mM NaCl, and the conversion color of paranitrophenyl phosphate was measured at 405 nm using a microplate reader (ELX808; BioTek).

### 2.5. ARS Staining

For ARS staining, cells were inoculated in a 24-well plate and cultured in OIM when the cells reached 70% confluence. OIM was cultured for 14 days, cells were washed with PBS three times andfixed in 4% paraformaldehyde for 20 min at RT. Washed with PBS for three times, calcium deposition was stained with Alizarin red staining (Cyagen) for 5–10 min at RT and then washed with PBS three times. The mineralized matrix stained with ARS was destained with 10% cetylpyridinium chloride for 1 hr at RT, the solutions were collected, then 200 *μ*L solutions were placed on a 96-well plate and read at 560 nm using a microplate reader (ELX808; BioTek).

### 2.6. MDC Staining

The presence of an autophagosome, as a marker of autophagy, was detected by the monodansylcadaverine (MDC) fluorescent dye as previously described by Ma et al. [21]. The autophagosomes were preferentially labeled by the lysosomptropic agent MDC, which can be incorporated into lipids in the autophagosome. Cells were seeded in 24-well culture plates and cultured in OIM for 3 days. Cells were rinsed in PBS (three times for 5 min), incubated with a 0.05 mM solution of MDC dye at 37°C for 30 min and then washed three times in PBS. Samples were observed under a fluorescence microscope (Leica, Wetzlar, Germany), with an excitation wavelength of 335 nm and an emission wavelength of 512 nm.

### 2.7. siRNA Transfection

Scrambled siRNA and YTHDF1 siRNA were designed and synthesized by GenePharma (Shanghai, China). The siRNAs were transfected into cells using siRNA-mate according to the manufacturer's instructions. The growth medium was changed 4 hr after transfection. Target RNAs and proteins were measured by qRTPCR 48 hr after transfection and Western blotting 72 hr after transfection, respectively. Sequences with a knockdown efficiency >70% were selected for the further experiments. Details of the sequences are listed in Table 1. On the 5th day of osteoinduction, siRNA was transfected again for a second knockdown.

### 2.8. Lentivirus Vector Infection

Lentiviruses overexpressing YTHDF1 were purchased from Beijing Tsingke Company and the viral titers were 1 × 10^8^ TU/mL. For infections, 40%–60% confluent hBMSCs were incubated with lentiviral particles in the presence of 5 *μ*g/mL polybrene. Infected cells were selected with 2 *µ*g/mL puromycin (Sigma–Aldrich) for 2 weeks. Stable clones were then maintained in 0.5 *µ*g/mL puromycin and observed and counted with a fluorescence microscope to ensure that the transfection rate was above 90% and YTHDF1 mRNA and protein levels were analyzed by qRT–PCR and Western blotting analysis, respectively.

### 2.9. RNA Extraction and qRT–PCR

Total RNA was extracted with the RNA isolation reagent (Takara) and RNA was quantified by a spectrophotometer at 260 nm wavelength (NanoDrop 2000; ThermoFisher). Total RNA was then reverse-transcribed with double-strand cDNA synthesis reagent (Takara) and cDNA (2 *μ*L) was used as the template for the qPCR reaction. The cycle conditions were as follows: 95°C for 30 s, followed by 40 cycles of 95°C for 5 s, and 60°C for 30 s. The 2^−*ΔΔ*CT^ method was used to calculate the relative gene expression level, with GADPH as the internal control. All primers used in this experiment were synthesized by Tsingke (Beijing, China) and are listed in Table 2.

### 2.10. Western Blot Analysis

Proteins were extracted from cells by lysing in RIPA buffer supplemented with protease inhibitors and phosphatase inhibitors. After protein quantification, cell lysates were suspended in 5x loading buffer and then equal amounts of proteins were subjected to migration on 4%–20% polyacrylamide linear gradient gels, then transferred to a polyvinylidene fluoride membrane (Millipore). The membranes were blocked by nonfat milk (5%) for 60 min and then incubated with primary antibodies overnight at 4°C. Horseradish peroxidase-conjugated goat antirabbit IgG (1 : 5,000, Beyotime) was used as a secondary antibody for 1 hr at room temperature. Immunoreactive bands were detected using an enhanced chemiluminescent detection reagent (Millipore). The signal intensity was measured using a Bio-Rad XRS chemiluminescence detection system (Bio-Rad, Hercules, CA, United States).

### 2.11. Immunofluorescence Analysis

Cells were cultured in induction medium in a 24-well plate and evaluated for RUNX2 and COL1A1 using a fluorescence microscope (EU5888; Leica) as follows. Cells were fixed in 4% paraformaldehyde for 15 min at room temperature, permeabilized, and blocked for 30 min in 0.3% Triton X-100, and 2% bovine serum albumin. Fixed cells were washed and incubated overnight at 4°C with anti-RUNX2 (1 : 400, Abcam) and anti-COL1A1 (1 : 400, Beyotime). Cells were incubated with a fluorescence conjugated secondary antibody (1 : 1,000, Beyotime) for 1 hr at 37°C, and nuclei were stained with 4′,6-diamidino-2-phenylindole (DAPI, Sigma–Aldrich) for 5 min. Samples were observed using an inverted fluorescence microscope (Leica).

### 2.12. Analysis of Public Databases

Gene expression profiles were downloaded from the GSE63591 [22]. The GSE63591 dataset contains ribosome profiling data from HeLa cells with YTHDF1 knockdown. A log2 ratio < −1 was used as a filter to identify differential transcripts. Gene Ontology (GO) and Kyoto Encyclopedia of Genes and Genomes (KEGG) analyses were performed with the Database for Annotation, Visualization, and Integrated Discovery (DAVID: http://david.ncifcrf.gov/) to identify significantly enriched pathways.

### 2.13. Cell Sheet Preparation

The cell sheets were fabricated in accordance with our previous studies [23]. In brief, confluent cells (5 × 10^5^/cm^2^) in flasks were cultured in MSC growth medium with the addition of vitamin C (20 *µ*g/mL) for 2 weeks to form a sheet of hBMSCs. The cells were then rinsed twice with PBS, and then detached intact from the substratum as cell sheets using a scraper.

### 2.14. Creation and Analyses of the Rat Tibial Fracture Model

Eight-week-old male Sprague–Dawley (SD) rats (∼200 g) were supplied by the Academy of Medical Sciences of Zhejiang Province. A rat tibial fracture model was created on the basis of a previous method [24, 25]. In brief, each rat was anesthetized by intraperitoneal injection of 0.3% pentobarbital sodium (30 mg/kg body). Exposed the right lower limb, made an incision lateral between the middle of the tibia tuberosity and the crest. An osteotomy with a transverse 1 mm wide from front to back was generated using an oscillating mini saw. An intramedullary needle (1.2 mm diameter stainless steel syringe needle) was inserted into the medullary cavity of the tibia through the patella tendon. The 15 tibial fractures in 15 rats were randomly allocated into three groups. In the blank group (*n* = 5), nothing was grafted onto the fracture site; in the NC group (*n* = 5), a lenticontrol BMSCs sheet was wrapped around the fracture site; and in the OE group (*n* = 5), a BMSCs sheet overexpressing YTHDF1 was grafted around the fracture site.

### 2.15. Radiographic Analysis

At 4 weeks postoperatively, five rats from each group were killed and the sample harvested in preparation for the radiographic and histological studies. Bone samples were examined with a *μ*CT-100 scanner (Scanco Medical, Switzerland) with a resolution of 24 *μ*m and analyzed using the accompanying supporting analysis and three-dimentional imaging software to evaluate callus formation. The bone volume fraction (BV/TV) and the trabecular thickness (Tb.Th) were calculated by standard three-dimensional microstructural analysis [25, 26].

### 2.16. Histological Evaluation

After micro-CT, all samples were decalcified using 10% EDTA (Sigma) with a solution change once a week for 6 weeks before embedding in paraffin. Serial sections 3 *μ*m thick were cut and mounted on polylysine-coated slides. Serial sections were deparaffinized and then stained with hematoxylin and eosin (HE), Masson's trichrome, Safranin O, and fast green according to standard procedure. The images were obtained using a microscope (Leica DM4000B; Leica, Wetzlar, Germany).

### 2.17. Statistical Analysis

All statistical analyses were performed with GraphPad Prism (version 8.0; GraphPad Software, San Diego, CA, United States). All experiments were carried out at least three times and the data are presented as means ± SD. The differences between two groups were analyzed using the Student's *t* test with two-tailed score. For comparisons between more than two groups, the one-way analysis of variance (ANOVA) followed by the Bonferroni post hoc test was used. In all analyses, *p* < 0.05 was taken to indicate the statistical significance.

## 3. Results

### 3.1. YTHDF1 Expression Is Upregulated during Osteogeneic Differentiation of hBMSCs

**Figure 1:**
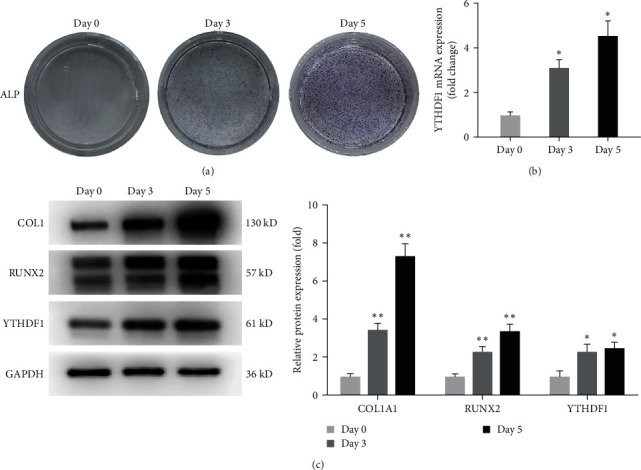
YTHDF1 was upregulated during osteogenic differentiation of hBMSCs. (a) Differentiation of hBMSCs into osteoblast by OIM and verification by ALP staining. (b and c) Relative mRNA levels and protein levels of YTHDF1 during osteoblastic differentiation on Day 0, Day 3, and Day 5.  ^*∗*^*P* < 0.05;  ^*∗∗*^*P* < 0.01 compared to the control group.

To explore the regulatory role of YTHDF1 in osteoblastic differentiation of hBMSCs, we first analyzed its expression. We cultured hBMSCs in OIM and confirmed osteoblastic differentiation of hBMSCs by ALP staining (Figure 1(a)). We examined the expression of YTHDF1 using qRT–PCR and Western blot analysis during osteoblastic differentiation of hBMSCs. Upregulated YTHDF1 was observed at both mRNA and protein levels compared to undifferentiated hBMSCs (Figures 1(b) and 1(c)). These results demonstrated that YTHDF1 expression levels are upregulated during osteoblastic differentiation of hBMSCs, which indicated that YTHDF1 could be involved in osteogenesis.

### 3.2. Downregulation of YTHDF1 in hBMSCs Impair Cell Proliferation and Osteogenic Differentiation

**Figure 2:**
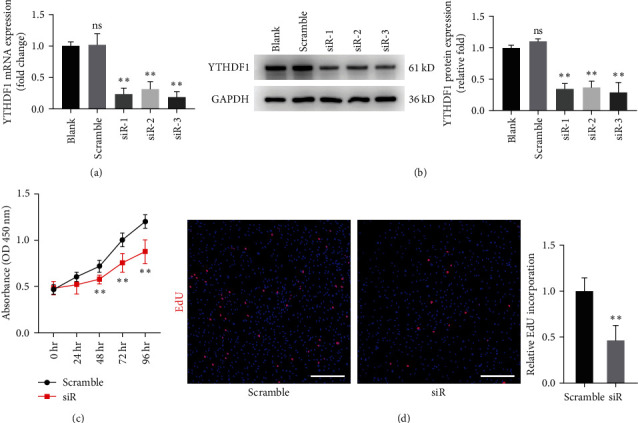
Knockdown of YTHDF1 impaired hBMSCs cell proliferation. (a) qRT–qPCR analysis of YTHDF1 on Day 2 after small-interfering RNA treatment. (b) Western blot analysis of YTHDF1 on Day 3 after small-interfering RNA treatment. (c) CCK-8 assays for siYTHDF1 transfected hBMSCs. (d) EdU-555 assays for siYTHDF1 transfected hBMSCs on Day 3. Scale bar: 200 *μ*m.  ^*∗*^*P* < 0.05 and  ^*∗∗*^*P* < 0.01 compared to the control group.

To investigate the biological function of YTHDF1 in hBMSCs, we designed (siRNA to knockdown the expression of YTHDF1 in hBMSCs. The inhibition was confirmed by qRT–PCR and Western blot analysis. The expression of YTHDF1 was downregulated by siRNA, especially siRNA3 (Figures 2(a) and 2(b)). Thus, siRNA3 was selected for subsequent experiment. The CCK-8 assay showed that silencing of YTHDF1 suppressed the proliferation of hBMSCs (Figure 2(c)). Additionally, a significant decrease was detected with the EdU assay of cells transfected with siYTHDF1 on Day 3, which was consistent with the results of the CCK-8 assay (Figure 2(d)).

**Figure 3:**
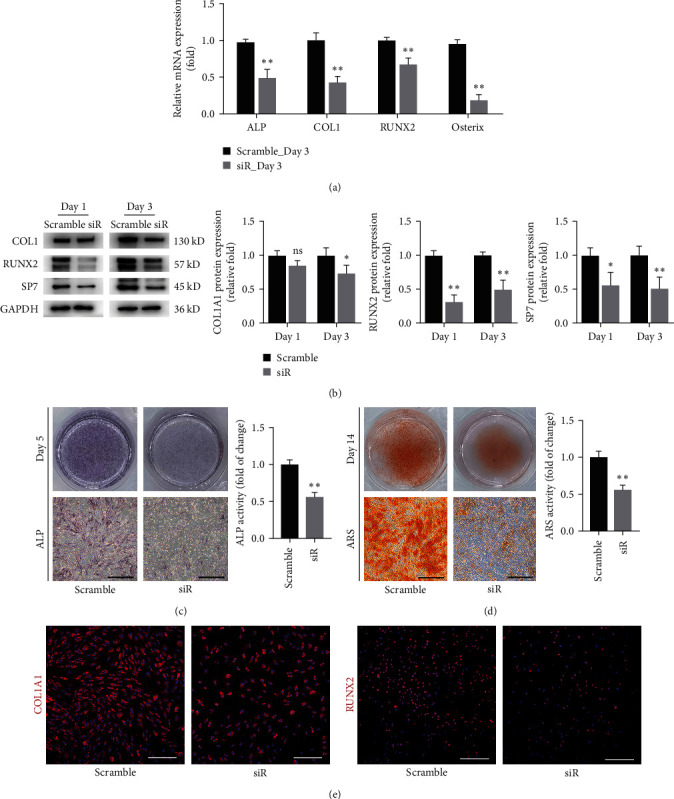
Knockdown of YTHDF1 compromised osteogenic differentiation of hBMSCs *in vitro*. (a) The expression of osteogenic markers (RUNX2, ALP, COL1A1, and osterix) was evaluted by qRT–PCR 3 days after osteogenic culture of hBMSCs knocked down by siYTHDF1. (b) Western blot analysis of osteogenic markers (RUNX2, COL1a1, and SP7) on Days 1 and 3 of osteogenic differentiation of hBMSCs. (c) Alkaline phosphatase activity was evaluated by ALP staining at Day 5 of osteogenic differentiation. Scale bar: 200 *μ*m. (d) Mineral deposits were determined by ARS staining on Day 14 of osteogenic differentiation. Scale bar: 200 *μ*m. (e) Immunofluorescence staining for the COL1A1 and RUNX2 protein after 3 days of osteogenesis. Scale bar: 200 *μ*m.  ^*∗*^*P* < 0.05 and  ^*∗∗*^*P* < 0.01 compared to the control group.

Osteospecific genes and proteins, including *ALP*, *COl1*, *RUNX2*, and *Osterix*, were determined by qRT–PCR and Western blot analysis. The results indicated that the mRNA levels of osteospecific markers were significantly reduced on Day 3 (Figure 3(a)). Western blot analysis revealed that the expression of COL1, RUNX2, and SP7 was also downregulated on Days 1 and 3 (Figure 3(b)). ALP staining and activity reduced on Day 5 after osteogenic induction (Figure 3(c)). ARS staining also showed that mineral deposits were significantly impaired in siYTHDF1 transfected cells (Figure 3(d)). Meanwhile, the immunofluorescence of COL1 and RUNX2 showed reduced expression in the siYTHDF1 group compared to the control group (Figure 3(e)). Taken together, our results suggest that YTHDF1 might play a regulatory role in BMSCs osteogenesis. However, more research is needed.

### 3.3. Upregulation of YTHDF1 in hBMSCs Promotes Cell Proliferation and Osteogenesis

**Figure 4:**
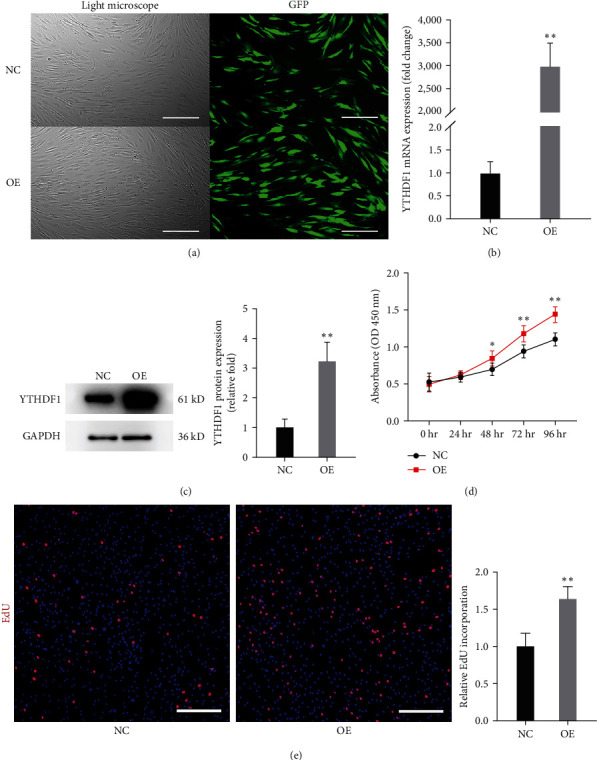
Upregulation of YTHDF1 promoted hBMSCs cell proliferation. (a) hBMSCs after lentiviral transfection and puromycin screening were observed under a normal microscope and a fluorescence microscope. Scale bar: 200 *μ*m. (b and c) qRT–PCR and Western blot analysis of YTHDF1 after lentiviral transfection. (d) CCK-8 assays for hBMSCs with YTHDF1 overexpression. (e) EdU assays for hBMSCs with upregulation of YTHDF1. Scale bar: 200 *μ*m.  ^*∗*^*P* < 0.05 and  ^*∗∗*^*P* < 0.01 compared to the control group.

Next, we used lentiviral vectors to establish hBMSCs overexpressing YTHDF1. The transfection rate was quantified by evaluating the ratio of cells positive for green fluorescent protein (GFP) to the total number of cells (Figure 4(a)). YTHDF1 expression was quantified by qRT–PCR and Western blot analysis (Figures 4(b) and 4(c)). The CCK-8 assay and the EdU assay showed that overexpression of YTHDF1 promoted hBMSCs proliferation (Figures 4(d) and 4(e)).

**Figure 5:**
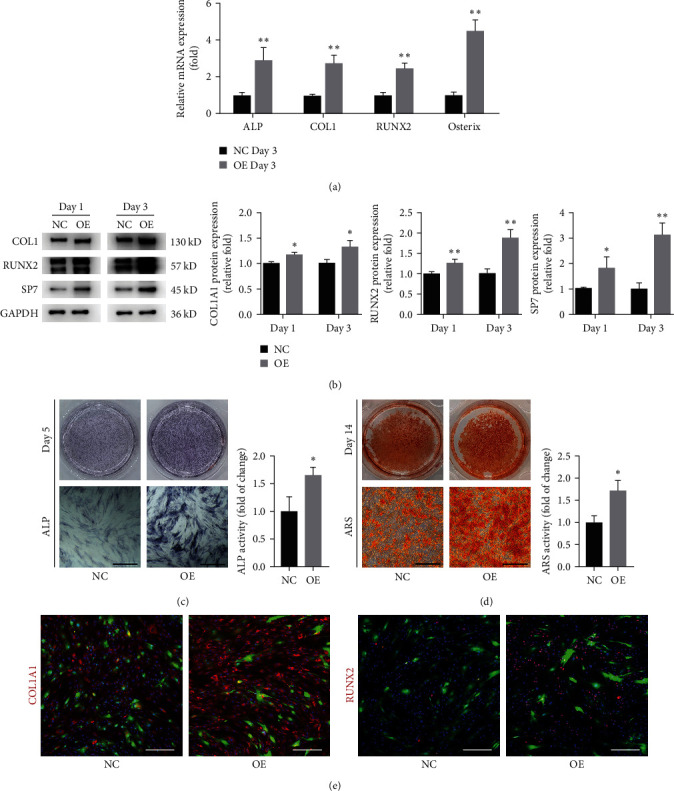
Upregulation of YTHDF1 promoted osteogenic differentiation of hBMSCs *in vitro*. (a) The expression of osteogenic markers (RUNX22, ALP, COL1A1 and osterix) was evaluated by qRT–PCR 3 days after osteogenic differentiation. (b) Western blot analysis of osteogenic markers after osteogenic differentiation for 1 and 3 days. (c) Alkaline phosphatase activity was evaluated by ALP staining at Day 5 of osteogenic differentiation. Scale bar: 200 *μ*m. (d) Mineral deposits were determined by ARS staining on Day 14 of osteogenic differentiation. Scale bar: 200 *μ*m. (e) Immunofluorescence staining for the COL1A1 and RUNX2 protein after 3 days of osteogenesis. Scale bar: 200 *μ*m.  ^*∗*^*P* < 0.05 and  ^*∗∗*^*P* < 0.01 compared to the control group.

We found that upregulation of YTHDF1 could elevate the expression of osteogenic markers at both mRNA and protein levels (Figures 5(a) and 5(b)). Meanwhile, upregulation of YTHDF1 significantly increased ALP activity and calcium mineralization (Figures 5(c) and 5(d)). The immunofluorescence of COL1 and RUNX2 also showed increased expression in the YTHDF1 overexpressing group (Figure 5(e)). Taken together, we conclude that YTHDF1 has a regulatory role in osteogenesis of hBMSCs.

### 3.4. YTHDF1 Increased Autophagy Levels during Osteogenesis of hBMSCs

**Figure 6:**
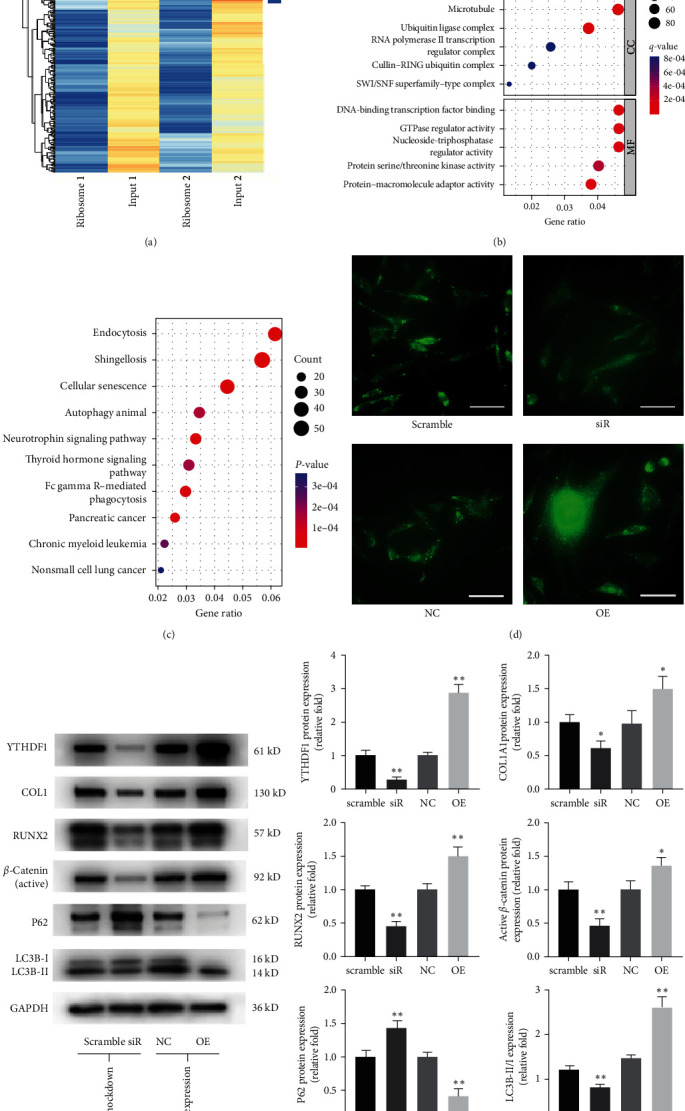
YTHDF1 increased autophagy levels in osteogenesis of hBMSCs. (a) Heatmap of differential translation efficiency in ribosome sequence of HeLa cells with YTHDF1 knockdown (GSE63591). A log2 ratio < −1 was used as a filter to identify differential transcripts. (b and c) GO and KEGG pathway analyses of YTHDF1 target transcripts. (d) MDC autophagosome staining after 3 days of osteogenesis. Scale bar: 50 *μ*m. (e) Protein levels were detected by Western blot analysis after downregulation or upregulation of YTHDF1 on Day 3 of osteogenic differentiation.  ^*∗*^*P* < 0.05 and  ^*∗∗*^*P* < 0.01 compared to the control group.

After revealing that YTHDF1 regulated the osteogenic differentiation of hBMSCs, the exact molecular mechanism remained to be explored. We analyze published ribosome profiling data from HeLa cells with YTHDF1 knockdown (GSE63591) [22]. Overall, the elimination of YTHDF1 resulted in 1,982 transcripts whose translational efficiency decreased over two times (Figure 6(a)). The gene ontology (GO) enrichment analysis showed that the biological process of these reduced transcipts is associated with regulation of the DNA metabolic process, proteasome-mediated ubiquitin-dependent protein catabolic process, ncRNA processing, DNA replication, and protein localization to the nucleus (Figure 6(b)). KEGG based functional gene analysis indicated that these transcripts are significantly enriched in some important pathways, such as “endocytosis,” “cellular senescence,” and “autophagy” (Figure 6(c)).

Recent evidence has shown that autophagy is a key regulator of bone metabolism [27]. A previous study also demonstrated that YTHDF1 promotes the translation of genes related to autophagy in HCC cells [19]. Considering that m6A modification is highly specific for cell types [6], we further detected autophagy levels in YTHDF1-silencing and YTHDF1-overexpressing BMSCs. MDC staining showed that YTHDF1 downregulation and upregulation significantly decreased and increased the number of MDC positive autophagosomes, respectively (Figure 6(d)). Meanwhile, Western blot analysis revealed that autophagy markers, LC3B Ⅱ/Ⅰ ratio was inhibited and p62 was activated by silencing YTHDF1 and vice versa (Figure 6(e)). With the data described above, we speculate that autophagy could contribute to the regulatory role of YTHDF1 during osteogenic differentiation of hBMSCs. Interestingly, WB analysis revealed that levels of active *β*-catenin was also significantly inhibited and increased in YTHDF1 knockdown and overexpression BMSCs (Figure 6(e)). Further investigation of the relationship between autophagy and YTHDF1 and osteogenesis of hBMSCs is required to elucidate the possible mechanism.

### 3.5. Upregulation of Autophagy Effectively Reverses Inhibition of Osteogenesis in YTHDF1-Knockdown hBMSCs

**Figure 7:**
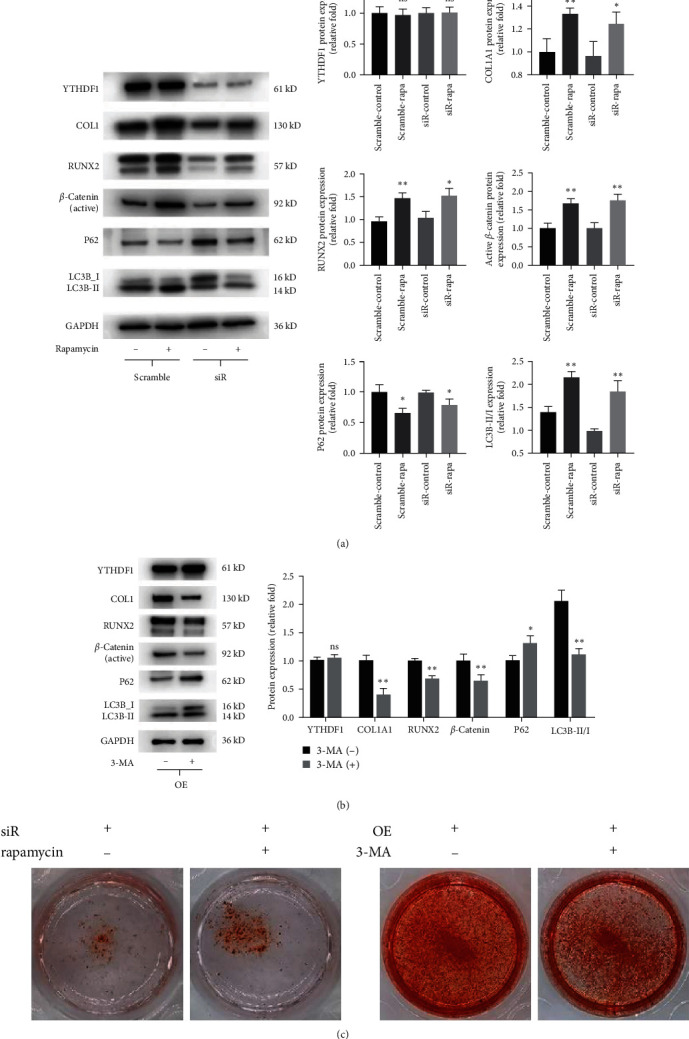
YTHDF1-regulated hBMSCs osteogenesis in part through the autophagy pathway. (a) Protein levels of hBMSCs transfected with siYTHDF1 after rapamycin treatment after osteogenic differentiation for 3 days. (b) Protein levels of hBMSCs overexpressing YTHDF1 treated with 3-MA on Day 3 of osteogenic differentiation. (c) Mineral deposits were determined by ARS staining on Day 14 of osteogenic differentiation.  ^*∗*^*P* < 0.05 and  ^*∗∗*^*P* < 0.01 compared to the control group.

As we observed that YTHDF1 increased autophagy levels during osteogenesis of hBMSCs, we next investigated the relationship between autophagy and YTHDF1 and osteogenesis of hBMSCs by manipulating autophagy levels in YTHDF1-silencing and YTHDF1-overexpressing hBMSCs. We used rapamycin (20 nM, 24 hr) as an inducer of autophagy to examine its effect on YTHDF1-silencing hBMSCs [28]. Autophagy activation was evidenced by accumulation of LC3B-Ⅱ and decreased p62 levels (Figure 7(a)). The cellular phenotypes showed that rapamycin effectively reversed YTHDF1 knockdown-induced inhibition of cell proliferation, as evidenced by the CCK-8 assay and EdU incorporation assay (Figure S1). Western blot analysis revealed that the expression of the ostegenetic markers COL1, RUNX2 was upregulated on Day 3. Consistently, the formation of mineralized nodules also increased on Day 14 (Figure 7(c)). Meanwhile, a significant upregulation of active *β*-catenin was observed in addition to the autophagy activation (Figure 7(a)).

### 3.6. Downregulation of Autophagy Impairs Osteogenesis in YTHDF1-Overexpressing hBMSCs

Next, the autophagy inhibitor 3-MA was used in YTHDF1-overexpressing hBMSCs to examine changes of cell proliferation and osteogenesis. A concentration of 5 mM recommended in the literature could effectively reduce autophagy [29]. The inhibition of autophagy was proved by the decrease in LC3B-Ⅱ levels and the accumulation of the p62 protein (Figure 7(b)). The CCK-8 assay and EdU incorporation assay showed that downregulation of autophagy impaired cell proliferation (Figure S2). We found that 3-MA could significanly inhibit the osteogenic differentiation capacity of YTHDF1 overexpressing BMSCs, as evidenced by the downregulated expression of osteogenic markers and impaired calcium mineralization (Figures 7(b) and 7(c)). In addition, we found that 3-MA could decreased active *β*-catenin levels in YTHDF1-overexpressing BMSCs, which showed a positive relationship. Together, these results verified that the effects of YTHDF1 on BMSCs osteogenesis are mediated by the autophagy pathway.

### 3.7. YTHDF1 Overexpressed Cell Sheets Accelerated Bone Healing in a Rat Tibial Fracture Model

To further assess the effect of YTHDF1 *in vivo*, a sheet of BMSCs with YTHDF1 overexpression was used in a rat tibial fracture model. The effect was confirmed by radiographic and histological analysis.

**Figure 8:**
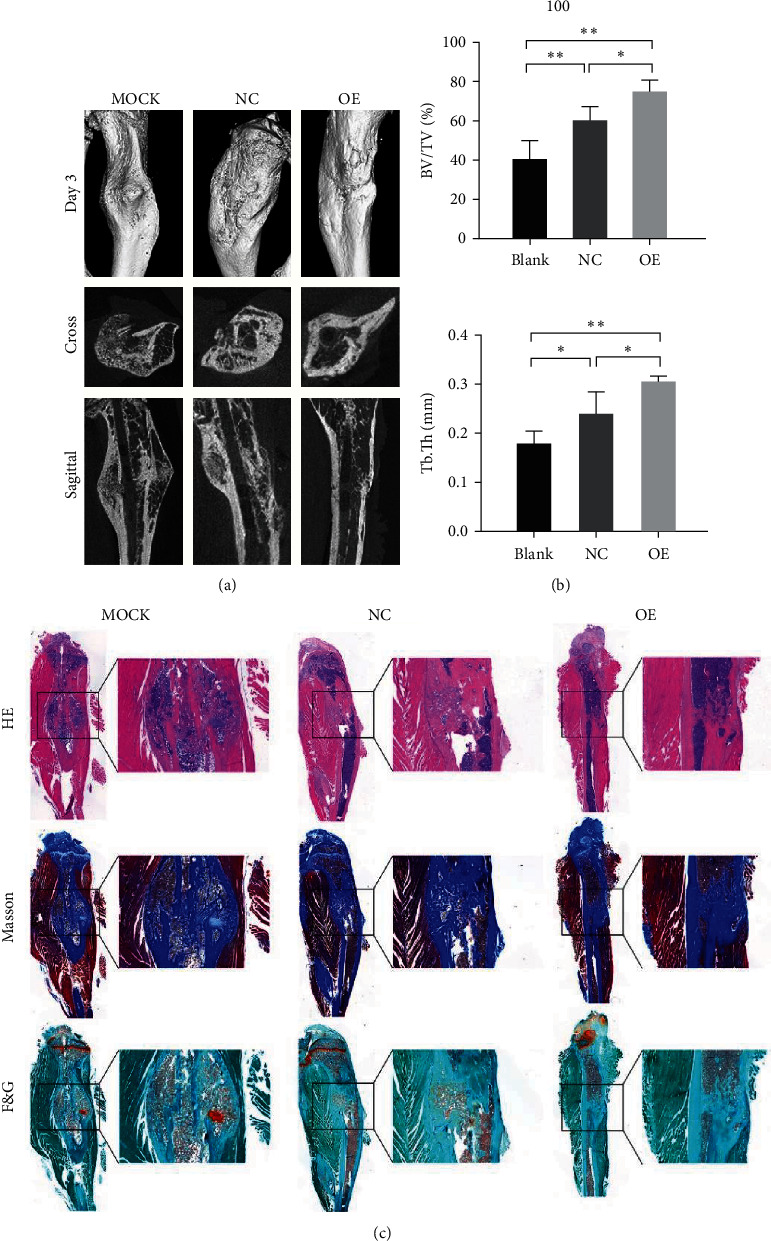
Cell sheets overexpressed with YTHDF1 accelerate bone formation in a tibial fracture model in rats *in vivo*. (a and b) Microcomputed tomography (microCT) analysis of bone healing. The bone volume fraction (BV/TV) and the trabecular thickness (Tb.Th) were analyzed by micro-CT. (c) Histologic analysis for bone healing. HE, hematoxylin and eosin staining; Masson, Masson trichrome staining; F&G, fast green and safranin staining. Magnification ×40.  ^*∗*^*P* < 0.05 and  ^*∗∗*^*P* < 0.01.

The results of sagittal reconstruction of the *μ*CT results showed that the fracture site was surrounding by mineralized callus in the blank group, characterized by woven bone and no obvious bony remodeling. In the NC group, some of the woven bone was placed by lamellar bone arranged in osteonal systems. In the OE group, the bony remolding was basically completed (Figure 8(a)). Quantitative analysis of micro-CT data demonstrated a significant increase in bone volume fraction (BV/TV) and Tb.Th in the NC and OE groups compared to the blank group. The increase was significantly greater in the OE group than in the NC group (Figure 8(b)).

Histological evaluation, including hematoxylin and eosin (HE), Masson's trichrome, safranin O, and fast green staining, revealed that the fracture site in the blank group was enveloped in a polymorphous mass of mineralized tissues consisting of woven bone and a few chondrocytes. In the NC group, cartilage in the callus was replaced by woven bone, and replacement of woven bone by lamellar bone was beginning. In the OE group, the remodeling of the callus was more complete, characterized by the replacement of woven bone with lamellar bone, indicating bone healing of the fracture (Figure 8(c)). The above results demonstrated that overexpressed YTHDF1 cell sheets accelerate bone formation *in vivo*.

## 4. Discussion

YTHDF1 is the most versatile and powerful reader protein of m^6^A-modified RNA. Work in the last decade clearly demonstrated that YTHDF1 plays a vital role in a multitude of biological processes through a stunning number of downstream targets [11–15]. Recently, YTHDF1 is suggested to affect osteogenic differentiation. Liu et al. [20] reported that YTHDF1 could facilitate BMSC osteogenesis through translation control of ZNF839. Moreover, the upregulation of YTHDF1 under hypoxia promotes osteogenesis of MC3T3-E1 cells via translational control of thrombospondin-1 [30]. However, because the differentiation of BMSCs is a complex and dynamic process, its exact role and molecular mechanism require further exploration.

Autophagy is a core molecular pathway for the preservation of cellular and organismal homeostasis [16–18]. Autophagosomes are the epicenter of autophagy due to their ability to encapsulate cytoplasmic contents targeted for lysosomal degradation [31, 32]. Emerging evidence shows that autophagy regulates bone metabolism [27–29]. Inhibition of autophagy accelerated bone loss, and activation of autophagy alleviated osteoporosis [29]. Furthermore, enhanced autophagy was found to significantly promote osteogenic differentiation and ameliorate adipogenic differentiation of BMSCs [33]. Intriguingly, m^6^A modification is known to exert effects on the regulation of autophagy in different disease backgrounds, including direct inhibition, the formation of autophagosomes, the initiation, and the enhancement of autophagy [34]. Additionally, previous study also indicated that YTHDF1 is involved in biogenesis of autophagy [19]. However, little is known about the interaction between YTHDF1 and autophagy during osteogenesis of BMSCs.

In our study, the relationship between YTHDF1 and autophagy was addressed. We found that overexpression of YTHDF1 enhanced autophagy levels, and downregulation of YTHDF1 reduced autophagy levels during osteogenensis of BMSCs, as evidenced by MDC staining and expression of autophagic proteins. These results revealed a strong and intricate relationship of YTHDF1 expression with autophagy levels. Therefore, we speculate that YTHDF1 could regulate osteogenetic differentiation of BMSCs by activating autophagy. The results of autophagy manipulation in both YTHDF1-silencing and YTHDF1-overexpressing BMSCs confirmed our speculations. As an autophagy inducer, rapamycin was used in YTHDF1-silencing BMSCs. The results showed that autophagy activation rescued cell proliferation and osteogenesis, as evidenced by CCK-8 assay, increased mineralized nodules formation and expression of osteogenetic markers. Meanwhile, inhibition of autophagy by 3-MA-reduced cell proliferation, the formation of mineralized nodules and the expression of osteogenetic markers in YTHDF1-overexpressing BMSCs. These results verified that the effect of YTHDF1 on the osteogenesis of BMSCs is mediated by the autophagy pathway.

The canonical Wnt/*β*-catenin signaling plays a pivotal role in promoting osteoblastic differentiation through its main effector, *β*-catenin [3]. A serious of studies revealed that autophagy could induce activation of Wnt/*β*-catenin signaling via autophagic degradation of specific pathway components [28, 35, 36]. Ge et al. [37] revealed that Wnt/*β*-catenin signaling pathway could be activated by autophagic degradation of APC during osteoblast differentiation. In this study, we found that YTHDF1 knockdown or overexpression significantly inhibited and improved the levels of *β*-catenin during osteoblastic differentiation of BMSCs, respectively. Moreover, our study also showed a positive relationship between autophagy and *β*-catenin expression. Based on these results, we inferred that YTHDF1 regulated osteoblastic differentiation of BMSCs via autophagy and *β*-catenin pathways. However, further research is required to elucidate its mechanisms.

In clinical settings, nonunion after a long bone fracture causes considerable morbidity when it occurs. Nonunion occurs in approximately 2% of all fractures, mainly due to impaired osteogenetic capacity [38]. Several reviews have emphasized the role of BMSCs in bone generation [39, 40]. Endogenous BMSCs, recruited from local soft tissue and bone marrow, migrate to the injury site, proliferate and differentiate into osteogenic cells. In this regard, the ability to enhance osteoblstic differentiation of BMSCs has generated much research interest in their use in the nonunion treatment [41]. In the present study, we conducted a bone fracture model and grafted with YTHDF1 overexpressing cell sheet. Consistent with *in vitro* data, we showed that fracture healing is accelerated with elevated bone volume fraction and Tb.Th. Our findings suggest a novel therapeutic target for bone repair and regeneration.

Collectively, our study demonstrated that YTHDF1 improved the proliferation and osteogenic differentiation of BMSCs through the autophagy and *β*-catenin pathway, revealing a previously unrecognized mechanism involved in the regulation of BMSCs osteogenesis by YTHDF1. Although further studies are warranted to address the adaptive role of YTHDF1 in skeletal metabolism, our findings suggest a novel therapeutic target for bone repair and regeneration.

## Figures and Tables

**Table 1:** Small-interfering RNA sequences to YTHDF1 and negative control.

	5′–3′
shRNA1	GGCGUGUGUUCAUCAUCAATT UUGAUGAUGAACACACGCCTT
shRNA2	CACCCAUAAAGCAUAACAUTT AUGUUAUGCUUUAUGGGUGTT
shRNA3	GGCUGGAGAAUAACGACAATT UUGUCGUUAUUCUCCAGCCTT
shRNA NC	UUCUCCGAACGUGUCAGGUTT ACGUGACACGUUCGGAGAATT

**Table 2:** Primers used for the analysis of mRNA levels by qRT–PCR.

Gene	Forward primer	Reverse primer
*YTHDF1*	5′-CGTGGACACCCAGAGAACAA-3′	5′-TGCCCAAAAACAGCATCGTG-3′
*ALP*	5′-GGGCATCATCCCAGTTGAGG-3′	5′-CACATATGGGAAGCGGTCCA-3′
*RUNX2*	5′-CGCCTCACAAACAACCACAG-3′	5′-ACTGCTTGCAGCCTTAAATGAC-3′
*Osterix*	5′-GTAGGACTGTAGGACCGGAG-3′	5′-ATAGTGAACTTCCTCCTCAAGCA-3′
*COL1A1*	5′-CTCGTGGAAATGATGGTGCT-3′	5′-ACCAGGTTCACCGCTGTTAC-3′
*GADPH*	5′-AATGGGCAGCCGTTAGGAAA-3′	5′-GCCCAATACGACCAAATCAGAG-3′

## Data Availability

The datasets used and/or analyzed during the current study are available from the corresponding author on reasonable request.

## Abbreviations

m^6^A: N6-methyladenosine
BMSCs: Bone marrow mesenchymal stem cells
CCK-8: Cell counting kit-8
qRT–PCR: Quantitative real-time PCR
MDC: Monodansylcadaverine
ALP: Alkaline phosphatase
ARS: Alizarin red staining
siRNA: Small-interfering RNA
3-MA: 3-Methyladenine
RUNX2: Runt-related transcription factor 2
GAPDH: Glyceraldehyde-3-phosphate dehydrogenase
COL1A1: Collagen type I alpha 1
Osx/SP7: Osterix.

## Supplementary Materials

Supplementary Materials Figure S1: Rapamycin as an autophagy inducer increased cell proliferation in YTHDF1-knockdownBMSCs. Scale bar: 200 *μ*m.  ^*∗*^*P* < 0.05 and  ^*∗∗*^*P* < 0.01 compared to the control group. Figure S2: Inhibition of autophagy reduced cell proliferation in YTHDF1-upregulated BMSCs. Scale bar: 200 *μ*m.  ^*∗*^*P* < 0.05 and  ^*∗∗*^*P* < 0.01 compared to the control group.
