# Supplementary material for: The m6A Reader YTHDF1 Accelerates the Osteogenesis of Bone Marrow Mesenchymal Stem Cells Partly via Activation of the Autophagy Signaling Pathway
Source: Stem Cells Int. 2023 Jul 25;2023:5563568. doi: 10.1155/2023/5563568 (PMC10393526; doi:10.1155/2023/5563568)
Supplement: Supplementary Materials — Figure S1: Rapamycin as an autophagy inducer increased cell proliferation in YTHDF1-knockdownBMSCs. Scale bar: 200 μm. ∗P < 0.05 and ∗∗P < 0.01 compared to the control group. Figure S2: Inhibition of autophagy reduced cell proliferation in YTHDF1-upregulated BMSCs. Scale bar: 200 μm. ∗P < 0.05 and ∗∗P < 0.01 compared to the control group. [file 5563568.f1.pdf]

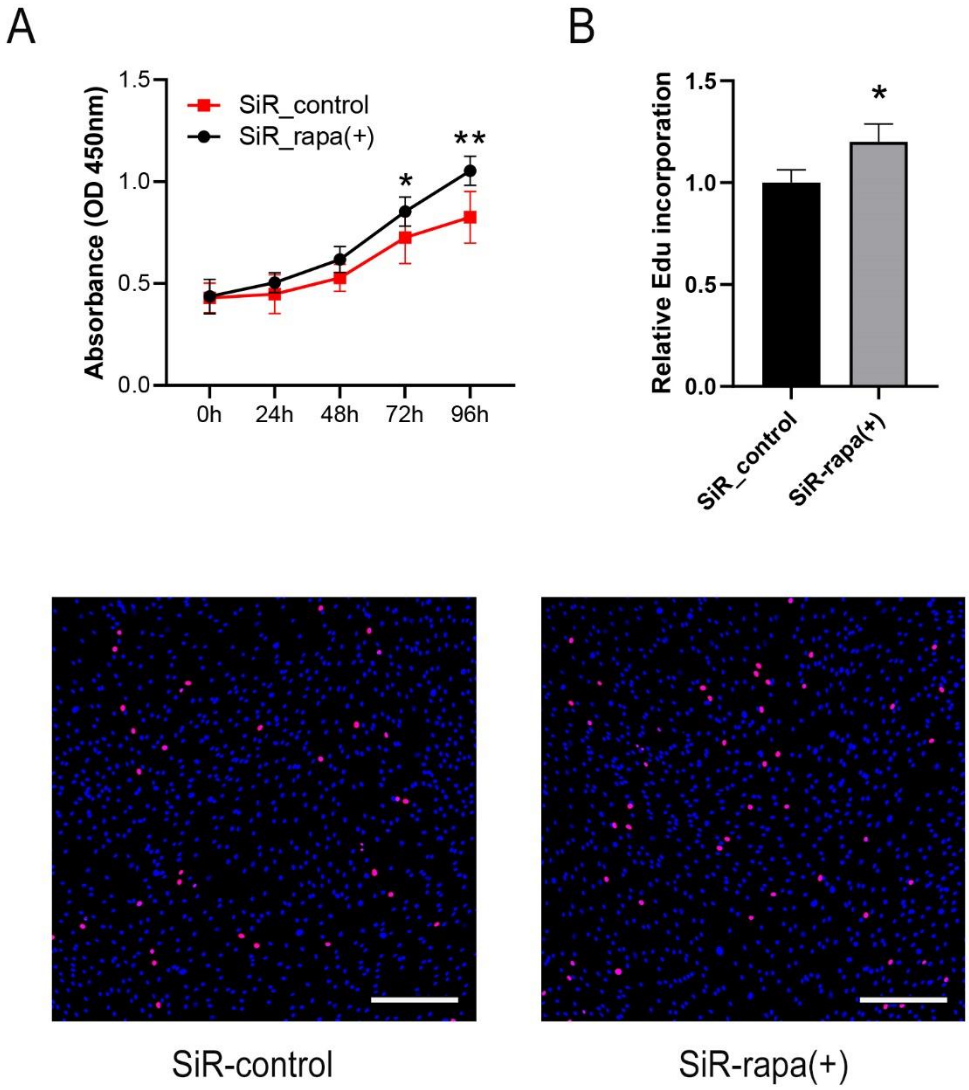

Supplementary FIGURE S1: Rapamycin as an autophagy inducer increased cell proliferation in YTHDF1-knockdownBMSCs. The CCK-8 assay (a) and EdU incorporation assay (b) of YTHDF1-downregulated BMSCs following rapamycin treatment on days 3. Scale bar: 200μm. **P* < 0.05, ** *P* < 0.01 compared to the control group.

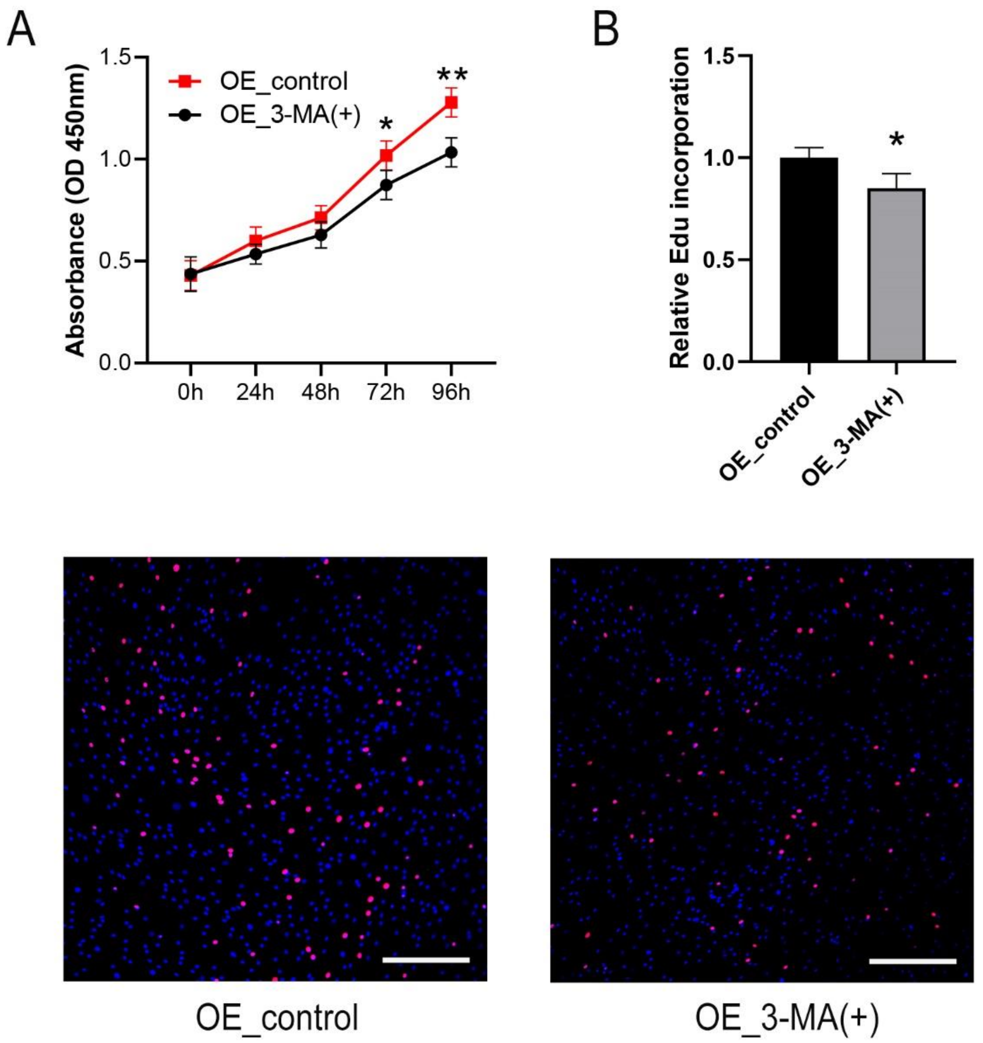

Supplementary FIGURE S2: Inhibition of autophagy reduced cell proliferation in YTHDF1-upregulated BMSCs. The CCK-8 assay (a) and EdU incorporation assay (b) of YTHDF1-upregulated BMSCs following 3-MA treatment on days 3. Scale bar: 200μm. *$P < 0.05$, ** $P < 0.01$ compared to the control group.
